# Impact of genetically modified Brinjal (Bt brinjal) on farmers’ income and production in Pabna District, Bangladesh

**DOI:** 10.1080/21645698.2025.2560698

**Published:** 2025-09-22

**Authors:** Md Amzad Hossain, Kazi Khalid Hossain, Niraj Prakash Joshi

**Affiliations:** a Social Innovation Science, Graduate Schol of Innovation and Practice for Smart Society, Hiroshima University (HU), Higashihiroshima, Japan; b Department of Economics, Gopalganj Science and Technology University, Gopalganj, Bangladesh; c Center for Peaceful and Sustainable Futures, The IDEC Institute, HU, Higashihiroshima, Japan; d International Economic Development Program, Graduate School of Humanities and Social Sciences, HU, Higashihiroshima, Japan; e TAOYAKA Program, HU, Higashihiroshima, Japan

**Keywords:** Bt brinjal, genetically modified crop, pesticides reduction, profit, propensity score matching, yield

## Abstract

Approval of Bt brinjal cultivation represents a crucial step forward for Bangladesh in agricultural biotechnology. However, the scalability of Bt brinjal adoption faces barriers, mainly due to resistance from traditional farmers, the limited evidence suggesting its socioeconomic impacts and full realization of its benefits. This study evaluates the socio-economic impacts of Bt brinjal adoption in the Pabna District. The study analyzed the impacts based on data from 489 brinjal farmers, comprising 197 adopters of Bt brinjal and 292 non-adopters employing propensity score matching, a method that helps to reduce selection bias in observational studies. The findings reveal that Bt brinjal adoption increased brinjal yield by 5,845.33 kg per hectare and raised profits by 226,577.54 BD taka (equivalent to 1,884.95 USD) per hectare. Additionally, pesticide costs were reduced by 41,269.499 BD taka (equivalent to 343.38 USD) per hectare. The increased yield and income and reduced use of pesticides demonstrate the economic and environmental advantages of Bt brinjal adoption. To harness the full potential of Bt brinjal, policymakers could adopt strategies that enhance farmers’ access to Bt brinjal technology and disseminate its positive socio-economic and environmental advantages through targeted educational programs. Such initiatives encourage widespread adoption and contribute to sustainable growth in the agricultural sector.

## 1. Introduction

Bangladesh, located on the South Asian subcontinent, stretches along the eastern shoreline of the Bay of Bengal and shares its northern and eastern borders with India and its southeastern border with Myanmar. Covering an area of 147,570 square kilometers, the country had a population of 167.6 million in 2020, with approximately 61.8% residing in rural areas.^1,2^ Notably, the agricultural sector remains a cornerstone of Bangladesh’s economy, particularly in addressing rural poverty and enhancing resilience to natural disasters. Indeed, agriculture contributed to 69% poverty reduction between 2005 and 2010 and 27% between 2010 and 2016.^3^ Although the sector’s share of GDP has declined as the country undergoes economic transformation, it still plays a crucial role in sustaining rural livelihoods, employing approximately 38% of the population.^4^ However, agriculture remains constrained by outdated practices, poor access to quality seeds and extension services, limited market connectivity, and technologies that often lack local relevance.^5^

Brinjal is a widely cultivated vegetable and a vital source of income for smallholders and resource-constrained farmers in Bangladesh. However, a significant limitation to successful brinjal production is infestation by eggplant fruit and shoot borer (EFSB), which causes significant yield and economic losses.^6^

**Figure 1.**
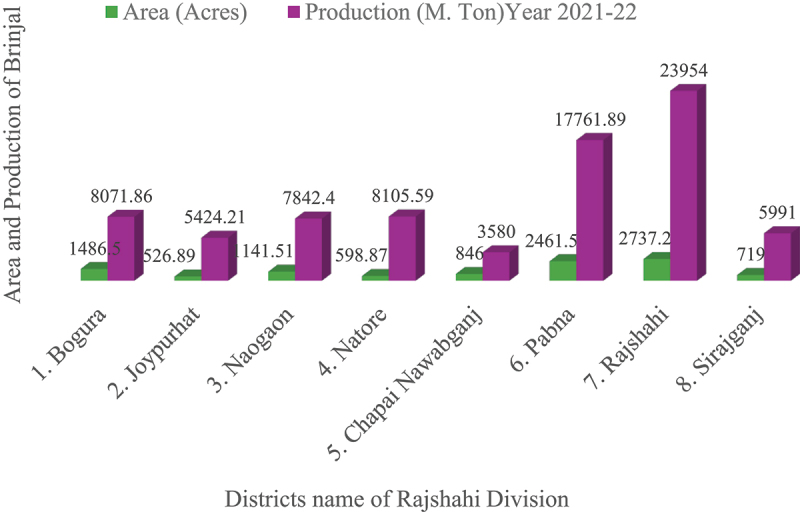
Area (Acres) and Production (M. Ton) of Brinjal production by District in Rajshahi Division (2021–2022). Source: BBS, 2023.

In the context of Brinjal cultivation during the agricultural year 2021–22 in Rajshahi Division, Pabna ranks second in total production, contributing 17,761.89 M.Ton from 2,461.50 acres of cultivated land, and demonstrates comparatively higher than districts such as Naogaon (7,842.40 M. Ton), Bogura (8,071.86 M. Ton), Natore (8,105.59 M. Ton), Sirajganj (5,991.00 M. Ton), Joypurhat (5,424.21 M. Ton), and Chapai Nawabganj (3,580.00 M. Ton) (Figure 1).^7^ Regarding production, brinjal is Bangladesh’s third most significant vegetable crop, with a cultivation area of approximately 50,956 ha during the 2016–2017 growing season.^8^ Brinjal *(Solanum melongena L.)*, commonly known as eggplant, is widely cultivated in Bangladesh and other parts of Asia year-round. However, the EFSB remains a significant challenge, causing widespread and chronic infestations that severely limit eggplant production.^9^ Consequently, brinjal cultivation suffers significant yield reductions, with losses attributed to EFSB (*Leucinodes orbonalis*) infestations reaching 86%.^10^ Moreover, farmers predominantly depend on the frequent application of chemical insecticides as the principal strategy for controlling EFSB damage and safeguarding crop productivity in Bangladesh.^10^

Pesticides play a crucial role in pest control globally, but their overuse and improper application can lead to significant risks because the annual consumption of over four million tons of pesticides and the availability of more than 1,000 products,^11^ the amount of pesticide use alone is not an adequate indicator of its impact. “Improper use” refers to practices such as over-application, misapplication, and use of non-targeted chemicals, all of which can harm human health, the environment, and biodiversity, contributing to pesticide resistance and ecosystem disruption. The demand for intensive farming practices largely drives this increasing reliance, and has been observed not only in developed countries, such as those in the European Union,^12^ but also in developing nations like Bangladesh.^13^ However, such widespread applications can lead to significant ecological damage, including harm to non-target organisms, disruption of food chains, and loss of biodiversity.^14,15^

The excessive use of pesticides is also a major and alarming problem in Bangladesh, and more so in the southwest, due to the cultivation of high-quality brinjals.^16^ Lack of technical knowledge and improper spraying have led to growers using excessive and unsafe amounts of pesticides on numerous occasions, resulting in low yields and inadequate pest control.^6,17^ Farmers apply pesticides 23–140 times during the cropping season to control diseases and infestations in brinjal crops, often without protective gear or adherence to safety measures.^18,19^ Proper pesticide application is essential for protecting farmers’ health and the environment. However, in Bangladesh, vegetable growers – despite their experience – still experience toxicity symptoms like headaches and vomiting.^20^ Additionally, the behavioral factors that influence their pesticide use are not well understood.^20^ The health and environmental hazards of the use of pesticides in vegetable cultivation, particularly for brinjal, remain a major and unexplored concern, even though studies have been conducted on the impact of pesticides on crop plants.^13^

In agriculture, practices such as improved breeding, agroecology, land management, and genetic modifications (GMOs) can contribute to sustainable increases in yield. GMOs have demonstrated great potential for enhancing crop yields, lowering greenhouse gas emissions, improving land-use efficiency, and lowering pesticide applications.^21^ The Bt brinjal was introduced in Bangladesh to combat the ever-present dangers of EFSB. A genetically modified brinjal crop carrying cry1Ac expresses an insecticidal protein from the soil bacterium *Bacillus thuringiensis* (Bt).^22^ To develop and adapt Bt brinjal technology, Cornell University collaborated with the Bangladesh Agricultural Research Institute (BARI) in 2005. Four open-pollinated varieties of Bt brinjal suitable for Bangladesh’s various agro-climatic zones have emerged as a result of such collaboration.^16,23^ Each cultivar was designated to enhance its adaptation to specific regions: ISD006 for Pabna and Chittagong, Kajla for Barisal, Nayantara for Rangpur and Dhaka, and Uttara for Rajshahi.^24^

In 2013, following nearly ten years of research, field trials, and regulatory assessment, Bangladesh approved the commercial cultivation of four Bt brinjal varieties, representing a significant advancement in the nation’s agricultural biotechnology.^22,25^ Despite this remarkable achievement, adoption at the national level has remained low. Moreover, despite their early involvement and potential, regions such as Pabna have had significantly low adoption and remain behind in the current literature. Of specific interest, the adoption rate plummeted to just 5.3% of the country’s brinjal cultivation land by 2023, from a peak of 11.9% in 2021.^26^ Nevertheless, empirical studies have consistently demonstrated that the Bt brinjal is highly effective in controlling EFSB infestations, significantly reducing pesticide use, improving yields, and lowering production costs, which are particularly advantageous for both the environment and resource-poor farmers.^10^

Although few studies have examined the adoption and impact of the Bt brinjal in Bangladesh, regions such as Pabna remain notably underexplored despite being an early pilot site. For instance, Ahmed et al.^18^ conducted a randomized controlled trial in Bogura, Gaibandha, Naogaon, and Rangpur, while Shelton et al.^6^ assessed the market value chain across Rangpur, Bogura, Rajshahi, and Jessore Tangail. The exclusion of Pabna from both the studies highlights a significant research gap. Moreover, the national adoption of Bt brinjals remains limited, potentially due to a lack of rigorous region-specific evidence regarding its broader socio-economic impacts. The existing literature often emphasizes yield gains and pesticide reduction but seldom addresses methodological challenges such as endogeneity or selection bias. To address this research gap, the present study applies the Propensity Score Matching (PSM) framework to evaluate the socioeconomic impacts of Bt brinjal adoption in Pabna, providing context-specific, policy-relevant insights into the role of genetically modified crops in enhancing agricultural outcomes in Bangladesh. To the best of our knowledge, this is the first study to apply the PSM approach to holistically evaluate the impact of Bt brinjal in the Pabna District of Bangladesh, specifically the Bt brinjal. The primary objective of this study was to assess the impacts of Bt brinjal cultivation on yield, profitability, and pesticide costs by comparing the outcomes of Bt brinjal adopting and non-adopting brinjal farmers in the Pabna District.

## 2. Methods

### 2.1. Study Area

**Figure 2.**
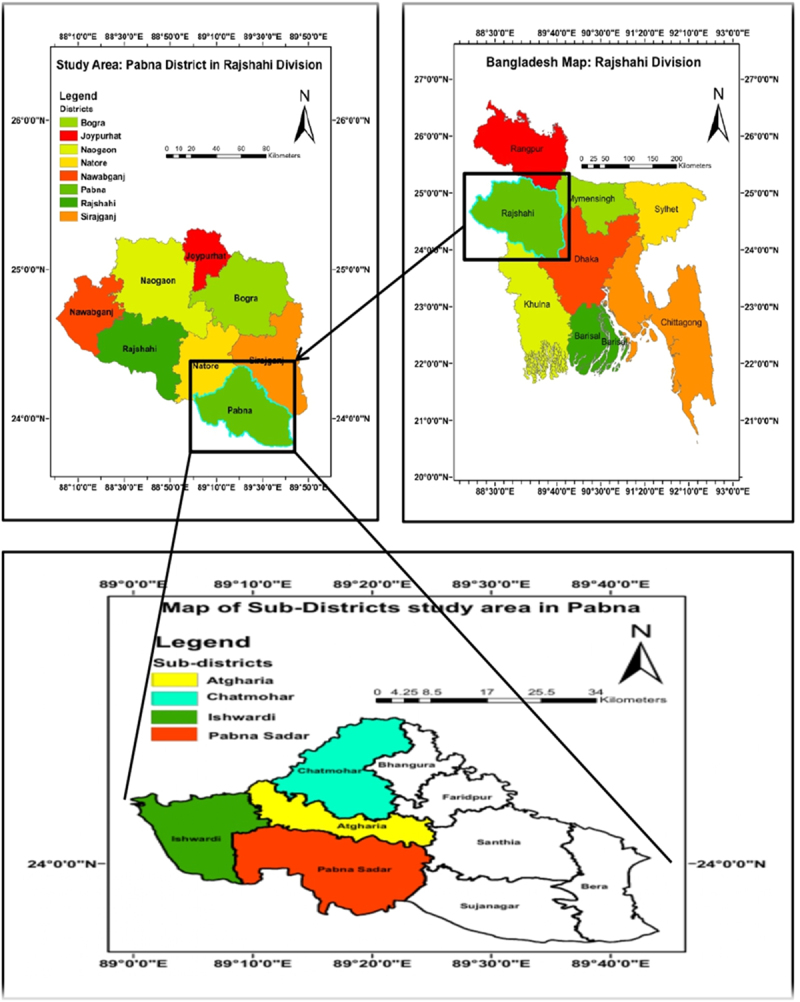
Pabna District in the Rajshahi Division of Bangladesh.

Bangladesh, with its 64 districts and 486 sub-districts,^7^ offers a detailed geographic setting for comprehensive production on brinjal. As a pioneering nation in South Asia for its cultivation, this study centers on Pabna District, renowned for extensive brinjal farming (Figure 2). Pabna is notable for its adoption of genetically modified Bt brinjal varieties, like BARI Bt Begun-4 (ISD 006), developed by the BARI. Its established agricultural practices and integration of modern technologies make it ideal for studying the economic and socio-environmental impacts of Bt brinjal cultivation. The district was chosen due to its high brinjal production, supported by data from agricultural authorities,^7^ which highlighted its importance in national crop output. In 2021–22, Pabna cultivated 2461.50 acres of brinjal, producing 17,761.89 metric tons, contributing significantly to Rajshahi Division’s overall production.^7^ This substantial output underscores Pabna’s crucial role in Bangladesh’s brinjal production. Focusing on Pabna, this study aims to examine the benefits of Bt brinjal farming within a region that exemplifies Bangladesh’s agricultural potential and environmental concerns.

### 2.2. Sampling and Sample Size

This study employed multistage sampling. In the first stage, the Pabna district was purposively selected because of its significant brinjal cultivation activity and agroecological diversity, making it suitable for analyzing differences in Bt brinjal adoption. This research was conducted in the Pabna district of Bangladesh, which includes four specifically chosen sub-districts: Pabna Sadar, Atgharia, Ishwardi, and Chatmohar, selected for their significant brinjal (eggplant) cultivation (Figure 2). Finally, the list of brinjal farmers was collected from the upazila agricultural office. The office provided a list of 530 brinjal cultivators and their contact information. We collected data from 489 farmers using a semi-structured questionnaire through face-to-face interviews, 41 farmers were unavailable during the survey period. For comparative analysis, the final sample was divided into two groups: the treatment group with 197 farmers cultivating Bt brinjals and the control group consisting of 292 farmers growing non-Bt brinjals. This sampling framework enabled a systematic assessment of the socio-economic, agronomic, and environmental effects associated with the adoption of genetically modified brinjal compared to traditional varieties.

### 2.3. Data Collection Technique

Data collection for this study took place from August 10 till September 20, 2024, using a semi-structured questionnaire. The questionnaire encompassed various topics, collecting demographic information, such as age, gender, education, and occupation of household members, as well as detailed land tenure information and brinjal production practices. Special emphasis was placed on input usage, particularly pesticide application, to assess the environmental impacts of Bt brinjal cultivation. Data were collected through face-to-face interviews conducted by a team of trained enumerators (20 members). These enumerators received careful training to maintain consistency and reliability in the data collection, ensuring high-quality and accurate information from every participants. Qualtrics was used for data collection. Qualtrics facilitates efficient data management and minimizes errors, thus enhancing the overall quality of the dataset.

### 2.4. Method of Estimation

PSM is widely used in observational research to estimate causal effects when randomization is not feasible. This refers to the conditional likelihood of receiving a specific intervention based on observed covariates.^27^ The estimated propensity score (PS) reflects the probability of treatment assignment given individual characteristics, enabling the construction of comparable groups for non-randomized analysis.^28^ Matching treated individuals with their untreated counterparts based on similar PS values ensures comparability between groups and helps control observed confounding factors.^29^ Once comparable groups are established, treatment effects can be estimated by directly comparing outcomes.^28–31^ Although traditional regression methods are commonly applied in such contexts, they may introduce bias when a covariate imbalance exists.^32^ PSM addresses this issue by balancing the distribution of covariates between the treatment and control groups, thereby improving the validity of causal inference.^32,33^ The implementation of PSM also requires methodological decisions regarding the matching algorithm, whether to allow replacement, and the choice of the matching ratio. While a 1:1 match is common, many-to-one matching is also frequently employed.^34^

This research employed PSM to reduce selection bias in observational data by simulating random treatment assignment through the pairing of treated and control groups.^35^ As the core methodology, PSM facilitates comparisons by aligning groups with similar propensity scores, thereby addressing selection bias.^36^ Estimation techniques, such as nearest neighbor matching (NNM) and caliper matching, enhance this process by minimizing discrepancies and establishing strict match thresholds, effectively replicating counterfactual outcomes.^37^ This rigorous approach allows a thorough evaluation of the impact of Bt brinjal on yield, profitability, and pesticide expenses.

Kernel matching, commonly used in PS analyses, was utilized to create a weighted average of control units and it assigns higher weights to observations closest to the treated units and gradually reduces the weight for more distant observations using a kernel function, as a result this approach improves the comparability between treatment and control groups in observational studies where randomization isn’t possible.^38^

On the other hand, in PSM, caliper matching restricts the formation of pairs to treated and control subjects whose propensity scores fall within a pre-defined tolerance (caliper width), ensuring that only sufficiently similar units are matched; this approach reduces confounding bias in observational studies, enhances match quality, and strengthens the assumption of common support.^39^ Mahalanobis Distance Matching (MDM) was used to balance multiple covariates by pairing treated units with control units through minimizing the Mahalanobis distance, ensuring the matches obtained were as close as possible.^40,41^

The estimation in PSM primarily focuses on the Average Treatment Effect on the Treated (ATET), which represents the average difference in outcomes for the treated group under treatment compared with a scenario without treatment.^32^ This study employs the ATET equation (Equation 1) to quantify the treatment effect.

(1) $$\mathrm{ATET}\left(q\right)=E\left[Y_{1}|T=1,B=q\right]-E[Y_{0}|T=1,B=q]$$

B denotes the vector of time-invariant variables used for matching. $E\left[Y_{1}|T=1,B=q\right]$ refers to the expected outcomes for units that have undergone treatment, whereas $E[Y_{0}|T=1,B=q]$ signifies the expected outcomes for the control units with similar vectors of time-invariant variables utilized for matching, i.e., B=q. Here, T is a binary variable indicating the use of BT brinjal or non-BT brinjal. To validate the findings, this study employed various matching methods, including NNM, Kernel Matching, Caliper Matching, and MDM. The thoroughness of this validation process instills confidence in the robustness of the study’s findings. This study utilized time-invariant variables that affect yield, profit, and pesticide costs in brinjal production as matching variables. These variables include gender, age, education, marital status, family size, total farm size, and farming experience. Besides land size under brinjal cultivation, membership of cooperatives and experience in brinjal farming were the matching variables (Table 1). All these factors are associated with outcome variables and treatment assignment. Similar pre-treatment variables have been employed in previous studies by Barslund and Tarp,^42^ Moahid et al.^35,43^ and Hossain & Joshi.^45^ (For more details about the PSM see the Table A2).

**Table 1.** Matching variables.

Variables	Descriptions
Gender	1 = Male, 0 = Female
Age	Age of the farmers in years
Education	Years of education of the farmers
Marital status	1 = Married, 0 = Otherwise
Family Size	Total number of family members
Total farm size	Hectares
Farming experience	Years
Land size under brinjal cultivation	Hectares
Membership in cooperatives	1 = Yes, 0 = No
Experience in brinjal farming	Years

Finally, the study incorporated an Inverse Probability Weighted Regression Adjustment (IPWRA). This robust technique combines propensity score weighting with regression adjustment to enhance the accuracy and consistency of the estimated treatment effects.^44,45^ Generally, IPWRA is considered a practical approach for mitigating potential biases that might arise from PSM estimations.^46^ Moreover, this approach integrates inverse probability weighting, which models treatment assignment with regression adjustment, and models’ outcomes to provide double robust estimates.^47^ The comprehensive nature of this analysis ensured that all aspects were considered, thereby enhancing the reliability of the results. Overall, IPWRA is a reliable method for estimating ATET, particularly in scenarios in which PSM may face issues of misspecification.^48^ These diverse methods collectively ensure the comprehensiveness and consistency of the findings. The IPWRA estimate was derived by applying the methodology detailed in Equation 2 below.

(2) $$\mathrm{ATE}T_{\mathrm{IPWRA}}=N^{-1}\sum_{i=1}^{n}\left[\left(\alpha_{1}^{*}+\beta_{1}^{*}X_{i}\right)-\left(\alpha_{0}^{*}+\beta_{0}^{*}X_{i}\right)\right]$$

The values of $\alpha_{1}^{*},\beta_{1}^{*}$ were determined using the methodology applied to the treatment groups. In contrast ($\alpha_{0}^{*},\beta_{0}^{*}$) are derived for the control group, as specified in the equations.

The estimation of ATET using PSM can be influenced by variables that are not directly observable or accounted for in the model. These unobservable variables may introduce biases that can affect the accuracy and reliability of the estimated effects, as highlighted in previous studies.^49,50^ Addressing these limitations is crucial to ensuring that the estimated treatment effect accurately reflects the causal relationship under investigation. The sensitivity test serves as the final step in assessing whether the results obtained from PSM are susceptible to the influence of unobserved variables.^32^

Li^32^ suggested three approaches for conducting sensitivity analysis: modifying the specification of the equation, utilizing instrumental variables, and applying the Rosenbaum Bounding (RB) test. This study applied the RB test to assess the sensitivity of estimated values. Assume that *w*_*1i*_ denotes the unobserved or latent characteristics associated with farmer i, which are not directly observed or measured, and is in the treatment group. and *w*_*0j*_ represent the latent features of farmer j, who is a control group member. The odds ratio will be.

(3) $$\Gamma =\exp\;(w_{1i}-\;w_{0j})$$

The Γ represents the odds of treatment. If the unobserved characteristics *w*_*1i*_ and *w*_*0j*_ are equal, it confirms that the treatment assignment is random. Consequently, the estimated ATET was neither overestimated nor underestimated, ensuring that the results were unaffected by hidden bias from unobservable variables.

## 3. Results and Analysis

### 3.1. Descriptive Statistics

A detailed analysis of the sociodemographic and agricultural characteristics of the sample population is presented in Table 2, providing a foundational basis for interpreting the determinants of brinjal production outcomes. The total sample includes 489 brinjal farmers from Pabna District, comprising both adopters and non-adopters of genetically modified Bt brinjal. Of these, 94% are male, with an average age of 45.12 years (SD = 8.69). The average year of education is 8.49 (SD = 4.21), and 95% are married. The typical household has 6.18 members (SD = 1.73). Regarding assets and experience, the average farm size is 0.81 hectares (SD = 0.51), and farmers have about 20.77 years (SD = 8.37) of farming experience. On average, 0.18 hectares (SD = 0.10) are used for brinjal cultivation. About 77% are members of agricultural cooperatives, and their experience specifically with brinjal farming averages 8.08 years (SD = 3.58).

**Table 2.** Descriptive Statistics.

Variables Names	Total Sample size (197+292=489)		Treatment Group (197)		Control Group (292)	
	Mean	SD	Mean	SD	Mean	SD
Gender (1 = Male, 0 = Female)	0.94	0.22	0.94	0.23	0.95	0.22
Age (Years)	45.12	8.69	44.62	8.08	45.45	9.07
Education (Years)	8.49	4.21	9.36	3.50	7.90	4.53
Marital status (1 = Married, 0= Otherwise	0.95	0.21	0.93	0.23	0.96	0.19
Family size (Numbers)	6.18	1.73	5.59	1.39	6.57	1.82
Total farm size (Hectare)	0.81	0.51	0.86	0.49	0.77	0.52
Farming experience (Years)	20.77	8.37	20.63	8.64	20.86	8.20
Brinjal land size (Hectares)	0.18	0.10	0.21	0.11	0.16	0.10
Membership in cooperatives (1 = Yes, 0 = No)	0.77	0.42	0.70	0.45	0.81	0.38
Experience in brinjal farming (Years)	8.08	3.58	7.77	3.46	8.28	3.65
**Outcome Variables**						
Yield (kg per hectare)	28922.89	4267.604	31889.36	3869.57	26921.54	3237.283
Profit (BDT per hectare)	470788.1	473620.9	581388.1	346363.6	396171	530568.5
Pesticide Cost (BDT per hectare)	10366.33	4694.57	7777.79	4071.07	12112.71	4266.098

Source: Field survey, 2024. Note: SD= Standard Deviation, BDT= Bangladeshi Taka.

The treatment group includes 197 farmers who have adopted Bt brinjal. Of these, 94% are male, with a mean age of 44.62 years (SD = 8.08). Their average formal education is 9.36 years (SD = 3.50). A high proportion of respondents (93%) are married, and the average family size is 5.59 members (SD = 1.39). The mean farm size is 0.86 hectares (SD = 0.49), and farmers have been engaged in general farming for about 20.63 years (SD = 8.64). Bt brinjal growers dedicate approximately 0.21 hectares (SD = 0.11) for brinjal cultivation. Among them, 70% are members of cooperatives, and their average experience in brinjal farming is 7.77 years (SD = 3.46).

The control group consists of 292 farmers who have not adopted Bt brinjal. In this group, 95% are male, and the average age is slightly higher at 45.45 years (SD = 9.07). The average educational attainment is 7.90 years (SD = 4.53), and 96% are married. Household size in the control group is larger, with an average of 6.57 members (SD = 1.82). The mean farm size is 0.77 hectares (SD = 0.52), and the average farming experience is 20.86 years (SD = 8.20). Farmers in this group dedicate an average of 0.16 hectares (SD = 0.10) for brinjal cultivation. Approximately 81% are members of cooperatives, and they report an average of 8.28 years (SD = 3.65) of experience in brinjal farming.

The comparison between the treatment and control groups highlights several notable differences. Bt brinjal adopters tend to be slightly younger and more educated than non-adopters, indicating a possible link between education and willingness to adopt agricultural innovations. Adopters generally have smaller family sizes, which may relate to household labor or demographic factors. They also tend to have larger farms and dedicate more land to brinjal cultivation than non-adopters, suggesting a higher capacity for commercial farming. Although adopters have slightly less experience in brinjal-specific farming, their overall farming experience is similar. Interestingly, non-adopters are more likely to be members of cooperatives, due to different social networks or institutional ties. These descriptive statistics offer a foundational understanding of group differences, forming the basis for further econometric analysis to assess the causal impact of Bt brinjal adoption.

This study examines outcome variables such as yield (kg per hectare), profit (Bangladeshi Taka -BDT per hectare), and pesticide costs (BDT per hectare). Bt brinjal adopters, on average, achieved a higher yield of 31,889.36 kg/ha compared to 26,921.54 kg/ha among non-adopters. Likewise, average profits were significantly greater for adopters (581,388.10 BDT per hectare) than for the control group (396,171.00 BDT per hectare). Conversely, pesticide expenses were considerably lower for adopters, with an average of 7,777.79 BDT per hectare versus 12,112.71 BDT hectare for non-adopters. These initial findings indicate clear productivity improvements and input cost reductions linked to Bt brinjal cultivation.

These sociodemographic and farming attributes provide a crucial analytical foundation for examining the interplay between household characteristics and brinjal production. These insights can guide the development of context-specific strategies to enhance productivity, strengthen farmer networks, and improve livelihoods in smallholder agricultural systems.

### 3.2. Impacts of Bt Brinjal Crops on Yield, Profit, and Pesticide Cost

The results in Table 3 demonstrate the significant impacts of Bt brinjal cultivation on yield, profit, and pesticide costs for farmers in the Pabna District of Bangladesh. The table presents the ATET using various estimation methods such as PSM, NNM, Caliper, Kernel, MDM, and IPWRA.

**Table 3.** Impacts of Bt brinjal on yield, profit, and pesticide costs.

Estimation method	Yield (Kg per Hectare)	Profit (BDT per Hectare)	Pesticide cost (BDT per Hectare)
	ATET	ATET	ATET
PSM	5845.33***(475.48)	226577.5***(87432.49)	−41269.49***(4073.73)
NNM	5845.33^***^(475.48)	226577.5***(87432.49)	−41269.49***(4073.73)
Caliper	5278.26***(353.67)	233554.6***(43900.54)	−46104.47***(2340.20)
Kernel	5589.09***(389.740)	224931.5***(51769.61)	−41723.75***(2767.23)
MDM	5872.12***(326.27)	306911.7***(50912.42)	−41617.78***(1452.17)
IPWRA	5584.93***(312.30)	231191.4***(38285.12)	77170.82***(1874.22)

Note: *** significant at the 1% level.

First, examining yield outcomes, all estimation methods consistently demonstrated a positive and statistically significant impacts of Bt brinjal cultivation. For instance, the PSM and NNM methods both report an ATET of 5,845.33 kilograms per hectare, with a high level of statistical significance (*p* < .01), indicating substantial improvements in yield. Other methods, such as Kernel and IPWRA, reveal significant yield benefits, with yields of 5,589.09 and 5,584.93 kilograms per hectare, respectively. These yield increases underscore the effectiveness of Bt brinjal in reducing losses from pest damage, primarily from eggplant EFSB, which hinders conventional brinjal farming.

The profitability analysis revealed similar positive outcomes. The PSM and NNM methods report a profit increase of 226,577.54 BDT per hectare, showing that Bt brinjal cultivation enhances yield and results in substantial profit gains for farmers. The MDM method even reported a higher profit increase of 306,911.73 BDT per hectare, further emphasizing these economic benefits. These findings align with those of previous studies highlighting the economic viability of genetically modified crops through reduced pest damage and improved yields.^16,18^

The analysis of pesticide costs reveals a critical aspect of the environmental and health impacts. Cultivating Bt brinjal led to a significant reduction in pesticide costs across all estimation methods. The PSM and NNM methods showed a cost reduction of 41,269.49 BDT per hectare (*p* < .01), indicating a substantial decrease in chemical pesticide usage, highlighting the potential of Bt brinjal to encourage more sustainable and environmentally friendly agricultural practices. Consistent reductions in pesticide costs are vital, as they suggest reduced exposure to harmful chemicals for farmers and local communities, along with decreased environmental pollution from pesticide runoff.

Overall, the findings in Table 3 are coherent and consistent across the various estimation methods, reinforcing the robustness of the results. Statistically significant improvements in yield, profit and substantial reductions in pesticide costs highlight the transformative potential of Bt brinjal cultivation. These outcomes demonstrate that genetically modified brinjals can benefit smallholder farmers in developing regions through yield improvement, profit increase, and pesticide cost reduction.

### 3.3. Balancing Property Between Treated and Control Groups

A comparison of the balancing properties between the treated and control groups, both before and after matching, as presented in Table 4, underscores the effectiveness of PSM on how the matching process mitigated biases, thereby enhancing the study’s capacity to make valid causal inferences regarding the impacts of Bt brinjal.

**Table 4.** Balancing property for treated and control group.

Variable	Matched	Mean Treated	Mean Control	% bias reduction	p>|t|
**Before Matching**					
Gender (1 = Male, 0 = Female)	U	0.94	0.94		0.96
Age (Years)	U	44.62	45.45		0.30
Education (Years)	U	9.36	7.90		0.00***
Marital status (1 = Married, 0 = Otherwise)	U	0.93	0.96		0.23
Family size (Numbers)	U	5.58	6.57		0.00***
Total farm size (Hectare)	U	0.86	0.77		0.06*
Farming experience (Years)	U	20.63	20.86		0.76
Brinjal land size (Hectares)	U	0.21	0.16		0.00***
Membership in cooperatives (1 = Yes, 0 = No)	U	0.70	0.81		0.00***
Experience in brinjal farming (Years)	U	7.77	8.28		0.11
**After Matching**					
Gender (1 = Male, 0 = Female)	M	0.94	0.94	−386.70	0.82
Age (Years)	M	44.62	45.06	47.80	0.61
Education (Years)	M	9.36	9.04	78.00	0.38
Marital status (1 = Married, 0 = Otherwise)	M	0.93	0.96	−31.00	0.14
Family size (Numbers)	M	5.58	5.48	89.70	0.46
Total farm size (Hectare)	M	0.86	0.85	89.10	0.84
Farming experience (Years)	M	20.63	20.99	−57.70	0.67
Brinjal land size (Hectares)	M	0.21	0.21	90.30	0.63
Membership in cooperatives (1 = Yes, 0 = No)	M	0.70	0.73	72.20	0.50
Experience in brinjal farming (Years)	M	7.77	7.41	30.20	0.27

Note: *** and * denote significance at the 1%, and 10% levels, respectively.

A balance check was conducted to evaluate the effectiveness of the matching process and to compare the covariate means between the treated and control groups before and after matching (Table 4). Prior to matching, several covariates demonstrated statistically significant differences, particularly education (*p* = .00), family size (*p* = .00), total farm size (*p* = .06), brinjal land size (*p* = .00), and membership in a cooperative (*p* = .00), indicating a substantial initial imbalance. After matching, the following variables became statistically insignificant: education (*p* = .38), family size (*p* = .46), total farm size (*p* = .84), brinjal land size (*p* = .63), and membership in cooperatives (*p* = .50), suggesting that matching successfully balanced these critical variables across groups. Moreover, farming experience, which was initially well balanced (*p* = .76), remained statistically insignificant after matching (*p* = .67). Minor imbalances persisted in variables, such as marital status (*p* = .14) and experience in brinjal farming (*p* = .27), although their bias was notably reduced. Overall, the substantial reductions in standardized bias, often exceeding 70–90% for key covariates, affirm the robustness of the matching procedure and the validity of subsequent treatment effect estimations.

### 3.4. Analysis of Sensitivity

The sensitivity analysis results in Table 5 comprehensively evaluate the robustness of the estimated effects of Bt brinjal cultivation on the yield, profit, and pesticide costs. Sensitivity analysis is essential for understanding the vulnerability of the study’s conclusions to hidden biases or unobserved confounding. The table reports the gamma (Γ) values alongside significance indicators (Sig+ and Sig-), revealing the stability of the treatment effects under various levels of potential unobserved biases. Overall, the sensitivity analysis demonstrates that the main findings of this study, namely, the positive impact of Bt brinjal on yield, profit, and pesticide cost reduction, are robust to potential unobserved biases. The consistency of the results across varying Gamma (Γ) levels underscores the reliability of the causal inferences drawn from propensity score matching analysis. These findings bolster the conclusions of the study and provide strong empirical support for promoting Bt brinjal as a sustainable agricultural practice in Bangladesh. The robustness of the results across key outcome variables ensures that policymakers and stakeholders confidently consider these benefits when promoting or expanding Bt brinjal cultivation.

**Table 5.** Sensitivity analysis.

Variable name	Gamma(Γ)	Sig+	Sig-
Yield (Kg per Hectare)	1.00	0	0
	1.05	0	0
	1.10	0	0
	1.15	0	0
Profit (BDT per Hectare)	1.00	0	0
	1.05	0	0
	1.10	0	0
	1.15	0	0
Pesticides Cost (BDT per Hectare)	1.00	0	0
	1.05	0	0
	1.10	0	0
	1.15	0	0

sig+ - level of upper bound significance (H_0_= ATET overestimated).

sig- - level of lower bound significance (H0 = ATET underestimated)

### 3.5. Assessment of Matching Quality

**Figure 3.**
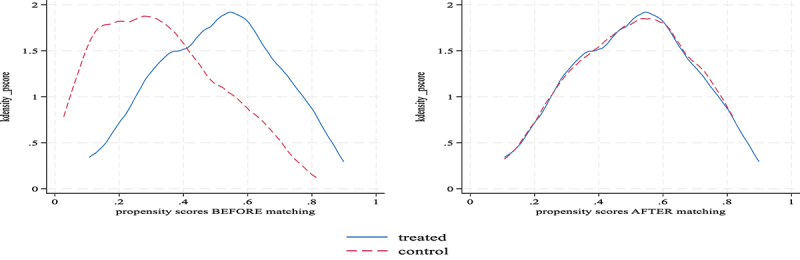
Propensity score distribution before (in left) and after matching (in right).

Figure 3 illustrates the distribution of the propensity scores for the treated and control groups before and after matching. The graph on the left shows the density of propensity scores before matching, whereas the graph on the right depicts the distribution after matching.

The left panel of the graph shows a significant imbalance between the treated (blue line) and control (red line) groups before the matching. The propensity scores of the treated group were skewed more toward higher values, while the control group’s scores were concentrated at lower values. This imbalance indicates that prior to matching, the probability distributions of receiving the treatment (in this case, adopting Bt brinjal) were markedly different between the two groups. Such a discrepancy suggests that simple comparisons between groups could lead to biased estimates of the treatment effect due to differences in covariates.

The right panel shows the success of propensity score matching in balancing the treated and control groups. Here, the density curves for both groups closely overlap, indicating that the distributions of the propensity scores have become much more similar. This overlap suggests that the matching process effectively adjusted for differences in observable characteristics between the treated and control groups, thus making them comparable.

**Figure 4.**
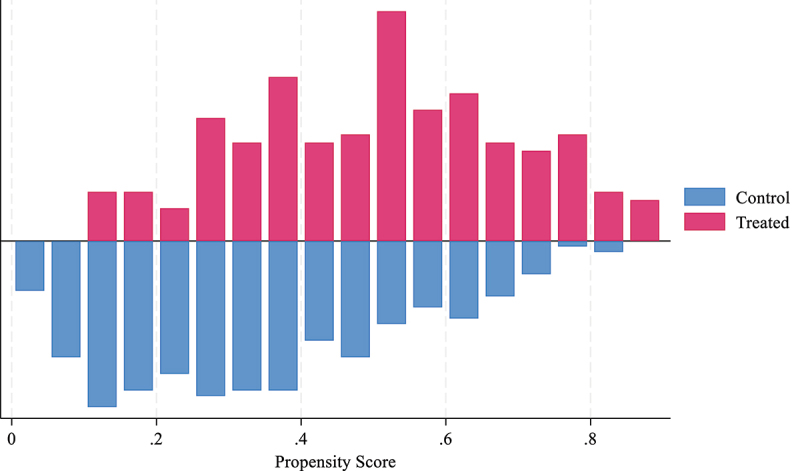
Common support.

The common support graph (Figure 4) illustrates the distribution of the propensity scores for the treated (pink) and control (blue) groups. A substantial overlap in the 0.2–0.9 range confirms the presence of common support, which is crucial for valid PSM. This overlap ensures that the treated and control observations are comparable, facilitating a more accurate estimation of Bt Brinjal’s effects on yield, profit, and pesticide use.

### 3.6. Test for Heterogeneity

Table 6 presents the subgroup-specific mean estimates of Bt brinjal’s impact on yield, profit, and production cost, categorized by cooperative membership status and landholding size. In the subgroup analysis, the results showed that farmers who are members of the cooperative are gaining higher profits compared to those who are not members of the cooperative. The results are significantly different, indicating a heterogeneous impact in terms of profit. Although several outcomes within each group showed statistically significant mean values (as indicated by “***”), the corresponding t-tests (see Table A1) revealed that most intergroup differences were not statistically significant. The distinction between groups is noted in cost savings between small and large landholders (*t* = 2.52, *p* < .05), indicating that Bt brinjal adoption leads to significantly greater cost reductions for large-scale cultivators. These results highlight the necessity of supplementing descriptive subgroup comparisons with formal inferential testing to ensure that the observed patterns reflect a statistically valid heterogeneity.

**Table 6.** Heterogeneity tests.

Outcome variables	Farmers with cooperative member	Farmers without members	Brinjal Cultivated land <0.18	Brinjal Cultivated land ≥ 0.18
Yield (Kg per Hectare)	5278.99*** (494.67)	5480.55*** (926.57)	5193.81*** (1021.40	5664.31*** (318.47)
Profit (BDT per Hectare)	188974.99*** (475.48)	141767.47 (138876.73)	220607.98*** (81842.34)	297654.16*** (128587.93)
Cost (BDT per Hectare)	−6035.62*** (715.63)	−6619.10*** (1452.34)	−5688.67*** (361.07)	−7635.13*** (697.73)

***, significant at the 1% level, and Sd errors are in parentheses. 1 USD = 119.51 Taka (01 September 2024).

## 4. Discussion and Implications for Policy

The current study revealed that cultivating Bt brinjal boosts yields by 5,278 to 5,872 kilograms per hectare using various estimation methods, indicating consistent and statistically significant enhancements. This is consistent with Ahmed et al.,^18^ who achieved a 51% yield improvement in a randomized controlled trial from the cultivation of Bt brinjal due to lower pest loss and improved retention after harvesting. Rashid et al.^51^ also observed no EFSB damage to Bt brinjal farmers, compared to 100% infestation in non-Bt. This broad variation supports the role of pest resistance in enhancing yield. Prodhan et al.^10^ also observed that EFSB infestation in Bt brinjal was below 5%, whereas infestation in control plots was up to 80%, resulting in higher gross returns and marketable yield. Shelton et al.^6^ recorded a 19.6% yield increase and found that 83.1% of Bt farmers were satisfied with their yield outcomes. Finally, the long-term field-level stability of the Bt trait was demonstrated by Prodhan et al.,^52^ who ensured that the EFSB populations in different districts remained Cry1Ac sensitive. Overall, evidence shows that Bt brinjal is responsible for increased yields, solely due to effective EFSB management; however, the extent of the gain depends on pest dynamics and management practices.

Profit returns from Bt brinjal cultivation in this study showed considerable increases, with thegains varying between 224,931 and 306,912 BDT per hectare based on the method of estimation, with the greatest returns seen when using MDM. These results are consistent with those of Ahmed et al.^18^ who reported that Bt brinjal farmers registered a 128% increase in net income, reduced pesticide expenditure, and increased marketable output. Rashid et al.^23^ also noted that Bt brinjal farmers earned 179,602 BDT per hectare, which is nearly six times higher than that earned by non-Bt brinjal farmers who earned 29,841 BDT per hectare. Moreover, Haque and Saha^9^ also stated that 72% of the surveyed farmers gained more income following Bt brinjal adoption and 28% of the farmers in their study did not gain greater profit due to low prices during selling, which negated the benefit of greater yield.

The pesticide spending findings in the present study revealed a considerable reduction across all estimation methods. These findings are supported by Ahmed et al.^18^ who noted a 37.5% lower pesticide expenditure in Bt brinjal cultivation, with fewer pesticide applications and lower pesticide toxicity. Specifically, they also attributed these reductions to improved health status in that Bt farmers were 11.5% points less likely to report symptoms of pesticide exposure. In the same way, Shelton et al.^6^ confirmed such trends, indicating that Bt brinjal farmers reported reduced application of insecticides and attributed this to improved health and environmental status. While indirectly not measuring pesticide cost savings or safety behavior, farmer perceptions strongly implied reduced chemical exposure and overall greater satisfaction. Rashid et al.^51^ also indicated that Bt brinjal farmers sprayed pesticides only 11 times per season compared to 41 applications by non-Bt farmers, representing a 61% reduction in pesticide spending. Haque and Saha^9^ documented that 77% of the farmers incurred reduced labor and chemical costs after Bt brinjal adoption. These findings suggest that, while Bt brinjal can cause substantial reductions in pesticide application and associated costs, the size of these benefits may depend on the occurrence of secondary pests and local pest management practices.

Extensive evidence shows that adopting Bt brinjal, a GMO crop, improves agricultural productivity by increasing yields, lowering production costs, and enhancing welfare for farm households, as seen in studies from Pakistan, India, and wider food security contexts.^53–55^ The current study’s findings on Bt brinjal in Bangladesh, indicating notable yield gains (5,845 kg per hectare) and profits (226,577 BDT per hectare), align with Sexton and Zilberman,^56^ who found that genetically engineered crops deliver significant yield and economic benefits across various settings. Additionally, our observation of reduced pesticide costs (approximately 41,269 BDT per hectare) supports the meta-analysis by Klümper and Qaim,^57^ which reported that the adoption of GM crops cuts chemical pesticide use by 37%, boosts yield by 22% and increases farmers’ profits by 68%. These consistent results highlight that Bt brinjal in Bangladesh not only boosts farm productivity and profitability but also offers environmental advantages, reinforcing global evidence of the economic and ecological gains from GM technologies. Policymakers should focus on expanding farmers’ access to Bt brinjal and promoting knowledge through targeted extension and training programs to accelerate adoption and foster sustainable agricultural growth.

Heterogeneity tests also identify farm size and membership in a cooperative as significant factors in enhancing Bt brinjal gains. Farmers who were cooperative members earned more profits. This finding implies that scaled and collective farming models are essential to maximize the economic and agronomic impacts of Bt brinjal cultivation.

Overall, this study confirms that the production of Bt brinjal significantly increases yield, improves farm profitability, and lowers pesticide costs for Bt brinjal farmers in the Pabna District. The positive impacts are firmly established using alternative estimation approaches and largely exist because of the effective management of the EFSB. While the findings are welcome, broader adoption will require overcoming the challenges related to market access. In the case of Bt brinjal, market access issues are often caused by limited infrastructure, lack of comprehensive market information, and price differences between traditional crops and genetically modified ones. Farmers growing Bt brinjal might struggle to reach local and national markets, leading to lower prices for their produce compared to non-GMO varieties. Moreover, some markets may resist genetically modified crops due to consumer concerns, and farmers could face challenges in developing dependable distribution channels. Nevertheless, Bt brinjal offers a promising way to achieve sustainable and economically viable eggplant farming in Bangladesh.

## 5. Conclusion

This study aimed to investigate the impact of genetically modified Bt brinjal cultivation on yield and profitability among farmers in the Pabna District of Bangladesh. The findings revealed a significant increase in brinjal yield of 5,845.33 kg per hectare and a rise in profits of 226,577.54 BDT per hectare, emphasizing the economic advantages of Bt brinjal cultivation. Furthermore, the study showed a decrease in pesticide costs of 41,269.49 BDT per hectare, illustrating both economic and environmental benefits. This study also emphasizes the need for policies supporting the adoption of genetically modified crops over larger areas and promoting cooperative farming models. Such policies could include assistance from cooperatives and incentives for sustainable practices that minimize pesticide reliance. By creating environments that facilitate these strategies, policymakers can help ensure significant improvements in agricultural productivity and sustainability, supporting economic resilience and rural development in agricultural communities.

Therefore, this study provides valuable evidence that supports and confirms previous research, offering a strong foundation for advancing the adoption of genetically modified crops within tailored agronomic frameworks. Future research should build on these findings and explore the long-term effects of such policies and their effectiveness in addressing the evolving needs of farmers in various agricultural environments.

Given these positive outcomes, policymakers should prioritize strategies to promote broader access to modern agricultural technologies such as Bt brinjal. The evidence from this study demonstrates clear economic benefits for farmers, including higher profits and reduced pesticide costs, while also contributing to environmental sustainability. However, challenges such as limited access, knowledge gaps, and regional disparities in adoption remain. Therefore, targeted policies aimed at improving access to information, supporting farmer education, and facilitating access to these technologies are crucial to ensure that more farmers can benefit from these innovations. Although this study was geographically limited to the Pabna district, its findings have implications for broader policy, particularly in the context of national food security and rural economic stability. Future research can explore these issues across diverse agricultural zones in Bangladesh to better inform comprehensive, region-specific national policy adjustments.

## APPENDICES

### Appendix-A

**Table A1.** T-Test for heterogeneous effects.

Outcome Variables	Coop member vs non-coop member (T-test)	Brinjal Cultivated land < 0.18 ha. vs Brinjal Cultivated land ≥0.18 ha (*T*- test)
Yield	− 0.19	− 0.44
Profit	0.34	− 0.51
Cost	0.36	2.52***

### Appendix-2

**Table A2.** Books on propensity score matching.

No.	Book Title	Authors/Editors	Publisher	ISBN/DOI
1	Propensity Score Analysis: Statistical Methods and Applications (2nd Edition)	Shenyang Guo, & Mark W. Fraser	SAGE Publications	Hardcover: 9781452235004 eBook: 9781483322520
2	Practical Propensity Score Methods Using R	Walter Laite	SAGE Publications	DOI: https://doi.org/10.4135/9781071802854.
3	Propensity Score Analysis: Fundamentals and Developments	Wei Pan & Haiyan Bai	The Guilford Press	ISBN-10: ‎ 1462519490 ISBN-13: ‎ 978–1462519491
4	Handbook of Matching and Weighting Adjustments for Causal Inference	José Zubizarreta, Elizabeth A. Stuart, Dylan S. Small & Paul R. Rosenbaum	CRC Press	ISBN-10: ‎ 0367609525 ISBN-13: ‎ 978–0367609528
5	Propensity Score Matching: When Units Meet	Marco Percoco	Springer	Online ISBN 978–3–319–09519–6

### Extra Articles/research papers on Propensity Score Matching method

#### Extra Link

1. Lunt, M. (2014). Selecting an Appropriate Caliper Can Be Essential for Achieving Good Balance with Propensity Score Matching. American Journal of Epidemiology, 179(2), 226-235. https://doi.org/10.1093/aje/kwt212.
2. Example 101.7 Mahalanobis Distance Matching. https://documentation.sas.com/doc/en/statug/latest/statug_psmatch_examples07.htm.
3. Ding, C., Li, Z., Ng, H. K., & Gao, W. (2025). Estimation of Treatment Effects based on Kernel Matching. *ArXiv*. https://arxiv.org/abs/2502.10958.
4. Komen, J. J., Belitser, S. V., Wyss, R., Schneeweiss, S., Taams, A. C., Pajouheshnia, R., Forslund, T., & Klungel, O. H. (2021). Greedy caliper propensity score matching can yield variable estimates of the treatment-outcome association – A simulation study. *Pharmacoepidemiology and Drug Safety*, *30*(7), 934-951. https://doi.org/10.1002/pds.5232.
5. Dai, Y., & Yan, Y. (2024). Mahalanobis balancing: A multivariate perspective on approximate covariate balancing. Scandinavian Journal of Statistics, 51(4), 1450–1471. https://doi.org/10.1111/sjos.12721.
6. Baltar VT, Sousa CA, Westphal MF. Mahalanobis’ distance and propensity score to construct a controlled matched group in a Brazilian study of health promotion and social determinants. Rev Bras Epidemiol. 2014 Jul-Sep;17(3):668-79. doi: 10.1590/1809 -4,503,201,400,030,008. PMID: 25272260.

## Appendix 3

Questionnaire

Serial no … … .

Date: ________________________

Dear Respondent, My name is HOSSAIN MD AMZAD, a master’s student at Hiroshima University. My research topic is “Impact of genetically modified brinjal crop cultivation on income and production quantity among farmers in Pabna District, Bangladesh.” I am going to conduct a survey focusing on the impact of genetically modified brinjal crop cultivation on farmers’ income and production quantity. The target of this survey is to collect information about farmers that will help to understand the impacts of genetically modified brinjal cultivation in Pabna District, Bangladesh. You were selected as one of the respondents to this study. I assure you that your identity will remain anonymous, and your answers will be used for academic purposes only. I am interested in your answers, responses, thoughts, and opinions on the following questions, which will be asked in the questionnaire. Please be frank and constructive in your responses based on your knowledge and personal experience.

Thanks for your participation.

Agree Not agree

Best regards,

Hossain Md Amzad

Researcher

e-mail: amzad15december@gmail.com

Mobile: 01717-432645

### Research Questionnaire On Impact Of Genetically Modified Brinjal Crop Cultivation On Income And Production Quantity Among Farmers In Pabna District, Bangladesh”

1. Id No-
2. Name –
3. District–
4. Sub-district (Upazila Name) name-
5. Mobile number:
6. Gender: 1 = Male, 0 = Others
7. Age: ______ years
8. Years of education: ______ years
9. Marital Status: 1 = Married, 0 = Others
10. Religion: 1 = Islam, 0 = Others
11. Family size: ______ number
12. Years of Farming Experience: ______ years
13. Membership in agricultural co-operatives: 1 = Yes, 0 = No
14. Total Farm Size: ______
15. Access to Agricultural Extension Services: 1 = Yes, 0 = No
16. If yes, Government = 1, Private = 0
17. What type of brinjal do you cultivate? 1 = GMO Bt Brinjal, 0 = non-GMO
18. If GMO, for how long ______ years
19. How many years have you cultivated Brinjal? ______ years
20. How many decimals of land did you cultivate with Bt. brinjal/______
21. How many decimals of land did you cultivate brinjal/______
22. How many kilograms of Bt. brinjals, did you produce last season/year? ______
23. How many kilograms of brinjals did you produce last season/year?
24. What was the average selling price per kg last season/year? ______ BDT
25. What was the total cost of your brinjal farming last season? ______ BDT
26. Pesticide cost ______ BDT
27. How many times did you apply pesticides during brinjal cultivation______
28. How much of your brinjal production is used for self-consumption? ______
